# Heart failure outcomes and empagliflozin effects in patients with heart failure and reduced ejection fraction in sinus rhythm or atrial fibrillation: Data from EMPEROR‐Reduced

**DOI:** 10.1002/ejhf.70021

**Published:** 2025-09-16

**Authors:** Michael Böhm, Javed Butler, Amr Abdin, Gerasimos Filippatos, João Pedro Ferreira, Stuart J. Pocock, Martina Brueckmann, Anne Pernille Ofstad, Elke Schueler, Christoph Wanner, Faiez Zannad, Stefan D. Anker, Milton Packer, on behalf of the EMPEROR‐Reduced Trial Committees and Investigators

**Affiliations:** ^1^ Klinik für Innere Medizin III, HOMICAREM (HOMburg Institute for CArdioREnalMetabolic Medicine), Universitätsklinikum des Saarlandes Saarland University Homburg/Saar Germany; ^2^ Department of Medicine University of Mississippi School of Medicine Jackson MS USA; ^3^ Baylor Scott and White Research Institute Dallas TX USA; ^4^ National and Kapodistrian University of Athens School of Medicine, Athens University Hospital Attikon Athens Greece; ^5^ Université de Lorraine, Centre d'Investigation Clinique‐Plurithématique Inserm CIC‐P 1433; Inserm U1116, CHRU Nancy Brabois, F‐CRIN INI‐CRCT (Cardiovascular and Renal Clinical Trialists) Nancy France; ^6^ RISE‐Health, Department of Surgery and Physiology, Faculty of Medicine University of Porto Porto Portugal; ^7^ Department of Medical Statistics London School of Hygiene & Tropical Medicine London UK; ^8^ Boehringer Ingelheim International Ingelheim Germany; ^9^ First Department of Medicine, Faculty of Medicine Mannheim University of Heidelberg Mannheim Germany; ^10^ Oslo Diabetes Research Center and Medical Department Boehringer Ingelheim Norway KS Asker Norway; ^11^ mainanalytics GmbH Sulzbach/Taunus Germany; ^12^ Department of Clinical Research and Epidemiology Comprehensive Heart Failure Center, University Hospital Würzburg Würzburg Germany; ^13^ Department of Cardiology (CVK) and Berlin Institute of Health Center for Regenerative Therapies (BCRT), German Centre for Cardiovascular Research (DZHK) partner site Berlin Charité Universitätsmedizin Berlin Berlin Germany; ^14^ Baylor University Medical Center Dallas TX USA; ^15^ Imperial College London UK

**Keywords:** Atrial fibrillation, Cardiovascular outcomes, Empagliflozin, Heart failure, HFrEF, Sinus rhythm

## Abstract

**Aims:**

Empagliflozin reduces cardiovascular death (CVD) or hospitalization for heart failure (HHF), slows estimated glomerular filtration rate (eGFR) decline and improves quality of life (QoL) in heart failure with reduced ejection fraction (HFrEF). Whether the effect of empagliflozin is consistent according to atrial fibrillation (AF) status is worth exploring.

**Methods and results:**

The impact of AF versus sinus rhythm (SR) on outcomes as well as on eGFR decline and QoL were studied post‐hoc in EMPEROR‐Reduced. Of patients with available rhythm analyses and after exclusion of patients with missing or paced rhythms, 2785 were included (AF, *n* = 928, SR, *n* = 1857). Differences were not significant for the primary endpoint (*p* = 0.66), first (*p* = 0.19) and recurrent HHF (*p* = 0.45). On placebo, alcohol consumption (interaction *p* = 0.32), body mass index (interaction *p* = 0.93), diabetes (interaction *p* = 0.52), hypertension (interaction *p* = 0.24) were not different between AF and SR. Low ejection fraction and high Kidney Disease: Improving Global Outcomes (KDIGO) class had higher event rates but without interaction between SR and AF, respectively. After a median follow‐up of 20 months, empagliflozin reduced CVD or HHF compared to placebo in AF and SR (hazard ratio [HR] 0.82, 95% confidence interval [CI] 0.63–1.08; and HR 0.69, 95% CI 0.56–0.84; interaction *p* = 0.29). The same applied to time to first HHF (interaction *p* = 0.20), while there was a borderline but insignificant interaction for first and recurrent HHF (*p* = 0.10). The effect on annual eGFR decline and QoL scores was not different. Incident AF was numerically lower but formally not significantly different (HR 0.66, 95% CI 0.40–1.09, *p* = 0.11, empagliflozin vs. placebo).

**Conclusions:**

In HFrEF, AF did not significantly modify outcomes after adjustment and did not associate with eGFR slopes. Empagliflozin reduced outcomes, eGFR decline and improved QoL regardless of AF or SR and probably reduced incident AF.

## Introduction

Atrial fibrillation (AF) is the most prevalent sustained arrhythmia associated with heart failure (HF).^1^ Risk factors such as alcohol consumption,^2^ overweight,^3^ diabetes,^4^ hypertension^5^ and chronic kidney disease^6^ are associated with high incidence and prevalence of AF. HF and AF are closely linked in their pathophysiology and outcomes and are characterized by a high comorbidity load,^7^ but whether AF modifies outcomes in HF with reduced ejection fraction (HFrEF) is less clear. Empagliflozin has been reported to reduce cardiovascular death (CVD) or HF hospitalization in HFrEF^8^ and HF with preserved ejection fraction (HFpEF).^9^ As some medications such as beta‐blockers act differently in HFrEF in AF versus sinus rhythm (SR),^10^ we studied the treatment effects of empagliflozin in patients with AF compared to SR on cardiovascular outcomes, estimated glomerular filtration rate (eGFR) decline and quality of life (QoL) and explored its effects on new‐onset AF in the database of the EMPEROR‐Reduced trial.

## Methods

### Study design

The design, baseline characteristics and results of the EMPEROR‐Reduced trial have been published previously.^8^ The ethics committees of each of the 622 participating institutions in 23 countries approved the protocol and all patients gave written informed consent. The registration identifier at ClinicalTrials.gov is NCT03057977.

### Study patients and procedures

Patients with HF and an ejection fraction of ≤40% were screened and those fulfilling eligibility criteria were randomized double‐blind in a 1:1 fashion to receive placebo or empagliflozin 10 mg daily in addition to their usual therapy. EMPEROR‐Reduced randomized patients with New York Heart Association class II–IV HF. Patients were required to have elevated N‐terminal pro‐B‐type natriuretic peptide (NT‐proBNP) at ≥600 pg/ml, ≥1000 pg/ml or ≥2500 pg/ml in those with an ejection fraction of ≤30%, 31–35% or 36–40%, respectively; these thresholds were doubled in patients with AF. Patients with an ejection fraction >30% could also be included if they had experienced a hospitalization for HF within the previous 12 months, irrespective of NT‐proBNP levels. Patients with or without diabetes were enrolled. During follow‐up, all accompanying treatments could be altered or initiated according to the changes in the clinical status of the patients at the discretion of the investigator. Patients were assessed at study visits for major outcomes, vital signs (eGFR by the Chronic Kidney Disease Epidemiology Collaboration equation), adverse events and changes in medications or clinical status that reflected changes in the course of HF.

All randomized individuals were followed up for the occurrence of prespecified outcomes for the entire duration of the trial regardless of whether the study participants had taken the study medication or were adherent with the study procedures according to the intention‐to‐treat principle. AF was defined as AF reported in any electrocardiogram before study treatment intake or history of AF reported as medical history. Patients with paced rhythms or unknown baseline rhythms were excluded from this analysis.

### Clinical outcome analyses

The primary endpoint of the composite of adjudicated CVD or hospitalization for HF was analysed as time‐to‐first event. The first secondary endpoint was the occurrence of all adjudicated hospitalizations for HF and the second secondary outcome was the rate of the decline in eGFR during double‐blind treatment (eGFR slope). Further endpoints assessed were the Kansas City Cardiomyopathy Questionnaire clinical summary score (KCCQ‐CSS) and new‐onset AF. Patients were grouped according to AF or SR. We evaluated the risk of HF events, CVD, eGFR decline and QoL in patients treated with placebo and empagliflozin according to heart rhythm at baseline. Thereafter, we compared the effects of empagliflozin within placebo on the above outcomes according to baseline rhythm. Finally, we compared the effects of empagliflozin versus placebo on the primary composite outcome according to the presence of the risk factors alcohol consumption, high body mass index, diabetes, hypertension, and extent of chronic kidney disease. Finally, we explored rates of incident AF among those with SR at baseline, and adverse events according to baseline rhythm.

### Statistical analyses

The effect of empagliflozin compared with placebo on the time‐to‐first event analyses was examined using Cox proportional hazard regression models with prespecified covariates of sex, geographical region, diabetes status at baseline, left ventricular ejection fraction, age and eGFR at baseline. The interaction between AF and SR and treatment group on the occurrence of the prespecified outcomes was tested using a treatment‐by‐rhythm interaction trend test. The first secondary outcome of total (first and recurrent) HF hospitalizations was evaluated with the use of the joint frailty model that accounted for informative censoring because of CVD. Between‐group differences in the slope of eGFR were analysed using a random slope model on on‐treatment data. Changes in KCCQ‐CSS were analysed in a mixed model with repeated measures. All models included the same covariates as the Cox model. The impact of individual additional risk factors on the primary endpoint were assessed within placebo patients using the same model with risk factor‐by‐rhythm interaction. Additionally, the impact of risk factors on the primary endpoint was assessed within placebo patients with an extended model using backward selection. The effect of empagliflozin compared to placebo within each category of the risk factors was assessed with the same model with treatment‐by‐rhythm interaction.

All analyses were performed by the sponsor, after agreeing on a statistical analysis plan using SAS version 9.4 (SAS Institute, Cary, NC, USA). All *p*‐values reported are two‐sided and *p* < 0.05 was considered as statistically significant in all cases. No adjustments for multiple testing were made due to the exploratory nature of the study.

## Results

### Patient characteristics

**Figure 1:**
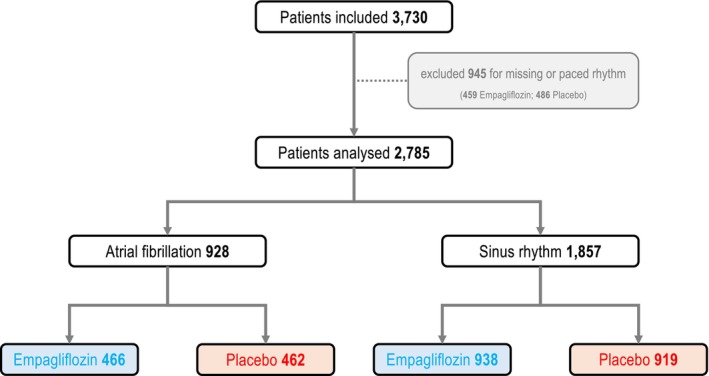
Flow of the predefined secondary analysis.

A total of 2785 patients were identified to have a complete analysis of the rhythm at baseline after exclusion of patients with paced rhythm. Among them, 928 were in AF and 1857 patients were in SR at baseline. Patients with unknown rhythm at baseline or paced rhythm were excluded from the analysis. The study flow is depicted in *Figure* *1*. More females were in SR than in AF, while more males exhibited AF at baseline. The baseline characteristics are shown in online supplementary *Table* *S1*. Patients with AF had a slightly different regional distribution with more patients with AF in Europe and less in Latin America. Baseline NT‐proBNP levels, heart rates as well as body weight and body mass index were higher in AF than in SR. Patients in AF had a lower eGFR and more often were in higher Kidney Disease: Improving Global Outcomes (KDIGO) risk categories, while baseline systolic blood pressure and baseline left ventricular ejection fraction were not meaningfully different.

### Association of baseline rhythm with outcomes

**Figure 2:**
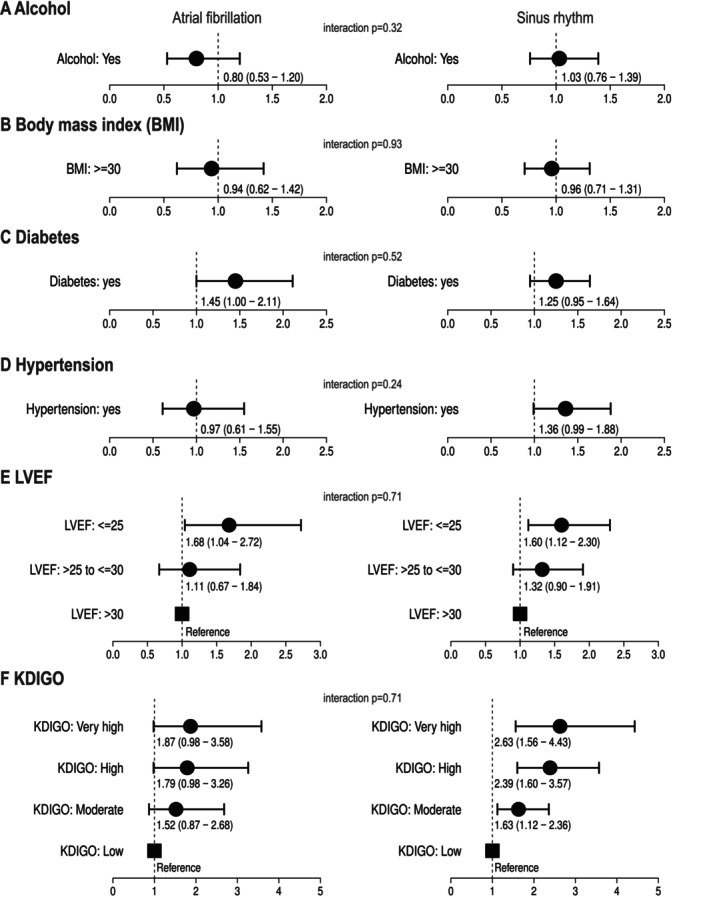
Impact of risk factors on time to first hospitalization for heart failure or cardiovascular death for (*A*) alcohol consumption (yes/no), (*B*) body mass index (≥30 or <30 kg/m^2^), (*C*) diabetes (yes/no), (*D*) history of hypertension (yes/no), (*E*) left ventricular ejection fraction (LVEF >30%, >25%–≤30%, ≤25%), (*F*) Kidney Disease: Improving Global Outcomes (KDIGO) risk group (very high, high, moderate, low) in atrial fibrillation and sinus rhythm within placebo patients. Reference group not shown for *A–D*.

On placebo, patients with AF had higher event rates of HF outcomes, but the differences were not significant (the primary outcome [*p* = 0.66], first [*p* = 0.19] and recurrent HF hospitalizations [*p* = 0.45]) when comparing within placebo patients. Patients with AF had lower KCCQ‐CSS values and similar declines of eGFR over time. We extended our assessment on patients by analysing the primary endpoint in presence of single risk indicators considering only patients on placebo (*Figure* *2*). There was no significant difference in risk by the presence of alcohol consumption (interaction *p* = 0.32, *Figure* *2A*), higher body mass index (≥30 kg/m^2^) (interaction *p* = 0.93, *Figure* *2B*), diabetes (interaction *p* = 0.52, *Figure* *2C*) and history of hypertension (interaction *p* = 0.24, *Figure* *2D*). Low ejection fraction and high KDIGO risk class conferred to higher risk but without interaction with AF and SR (interaction *p* = 0.71 for both, *Figure* *2E,F*). Using backward selection, only KDIGO risk class and left ventricular ejection fraction showed a significant impact on the risk of the primary outcome without an influence on the difference between AF and SR (data now shown).

### Effect of empagliflozin on efficacy outcomes according to atrial fibrillation or sinus rhythm

**Figure 3:**
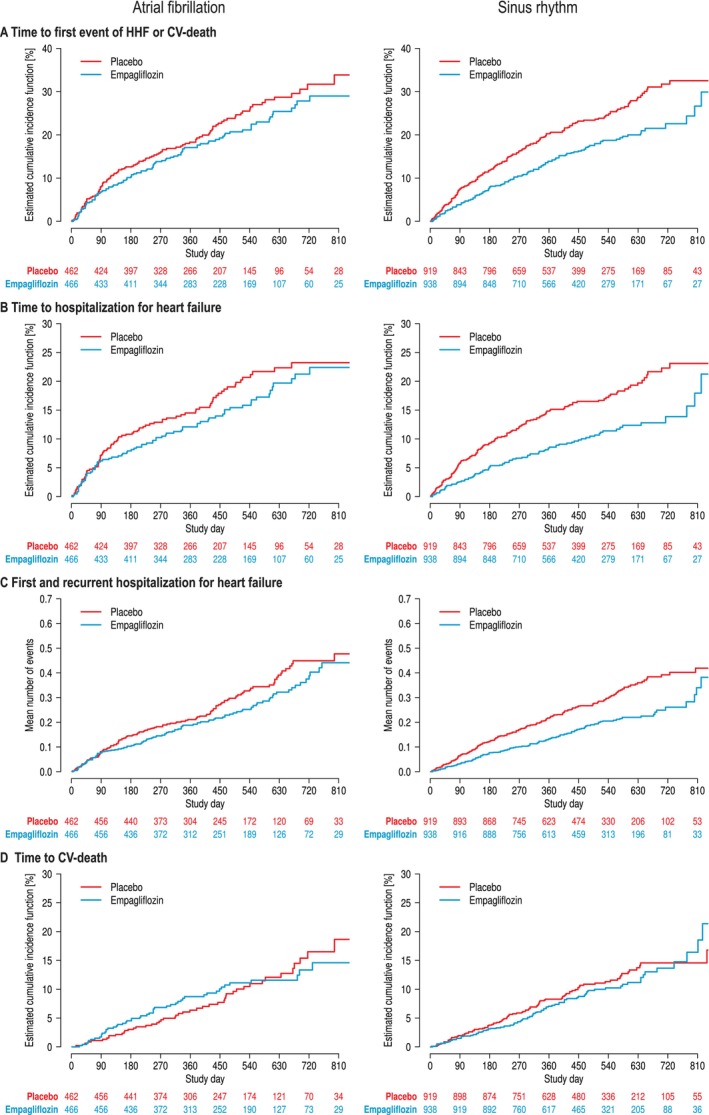
Cumulative incidence curves for empagliflozin versus placebo by atrial fibrillation or sinus rhythm. Cumulative incidence function of (*A*) the primary outcome (composite of first hospitalization for heart failure [HHF] or cardiovascular [CV] death), (*B*) first HHF, (*C*) first and recurrent HHF (mean cumulative function) and (*D*) CV death. Data adjusted for competing risk by death types, which were not part of the endpoint under investigation (i.e. all‐cause death for HHF).

**Figure 4:**
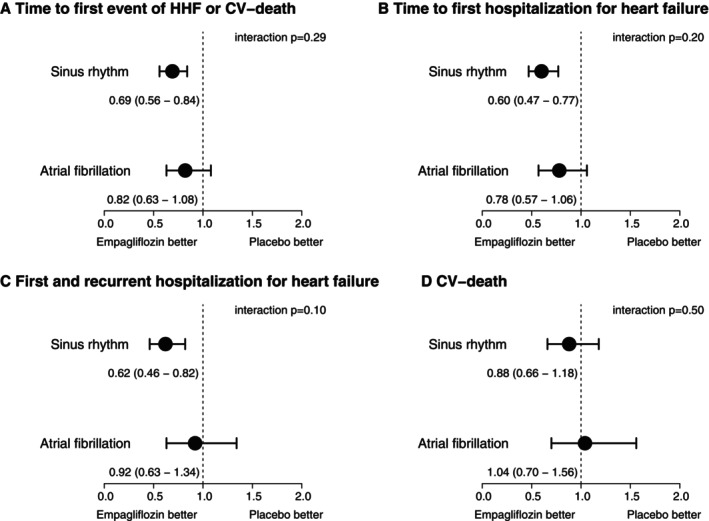
Empagliflozin effects in sinus rhythm and atrial fibrillation for (*A*) time to first event of adjudicated hospitalization for heart failure (HHF) or cardiovascular (CV) death, (*B*) time to first HHF, (*C*) first and recurrent HHF, and (D) CV death. [Correction added on 3 October 2025, after first online publication: Figure 4 has been corrected in this version.]

*Figure* *3* summarizes cumulative incidence curves for the primary endpoint, time to first hospitalization of HF, first and recurrent hospitalization for HF (mean cumulative incidence) and CVD. The relative risk reduction for the primary outcome by empagliflozin was similar between AF and SR (*Figure* *3A*) (primary endpoint interaction *p* = 0.29). Similar results were observed for first adjudicated hospitalization for HF (*Figure* *4B*) (interaction *p* = 0.20). A reduction of first and recurrent hospitalization for HF was more clear in SR (hazard ratio [HR] 0.62, 95% confidence interval [CI] 0.46–0.82) than in AF (HR 0.92, 95% CI 0.63–1.34), but formally not significant (interaction *p* = 0.10) (*Figure* *4C*). Empagliflozin did not reduce CVD, which also had no interaction between SR and AF (interaction *p* = 0.50) (*Figure* *4D*). There was no effect of empagliflozin on all‐cause mortality (not shown).

### Effects on estimated glomerular filtration rate decline

**Figure 5:**
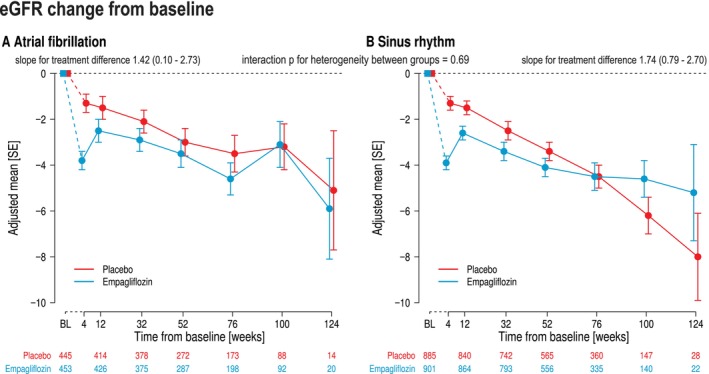
Estimated glomerular filtration rate (eGFR) adjusted mean difference (ml/min/1.73 m^2^) change over time in patients treated with empagliflozin or placebo in patients with (*A*) atrial fibrillation or (*B*) sinus rhythm. eGFR was determined by using the Chronic Kidney Disease Epidemiology Collaboration equation. No corrections for multiple testing were applied. SE, standard error.

Empagliflozin reduced the slope of eGFR decline from week 4 to the end of follow‐up (*Figure* *5*). Overall, the difference of mean slope of decline compared to placebo was similar between AF and SR (interaction *p* = 0.69).

### Effects on quality of life

**Figure 6:**
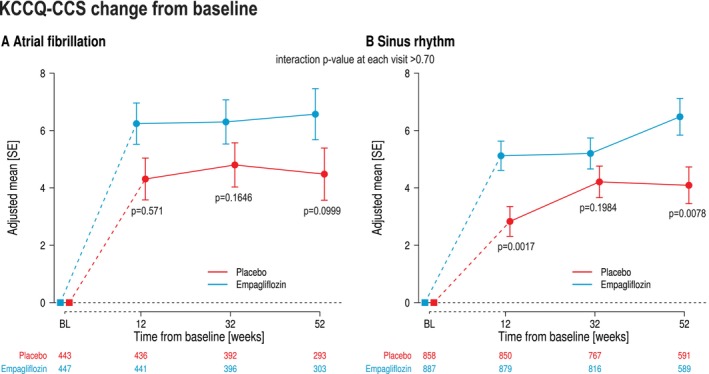
Effect of empagliflozin and placebo in patients with (*A*) atrial fibrillation and (*B*) sinus rhythm. No corrections for multiple testing were applied. KCCQ‐CSS, Kansas City Cardiomyopathy Questionnaire clinical summary score; SE, standard error.

The mean change in KCCQ‐CSS by treatment arms over time is presented in *Figure* *6*. Compared to placebo, patients treated with empagliflozin showed a meaningful improvement in mean KCCQ‐CSS with no significant difference between AF and SR (interaction *p*‐value at week 52 = 0.84). The responder analysis showed that patients in the empagliflozin arm were more likely to show an improvement >5 points and were less likely to show a deterioration in both AF and SR (data not shown).

### Time to new‐onset atrial fibrillation

**Figure 7:**
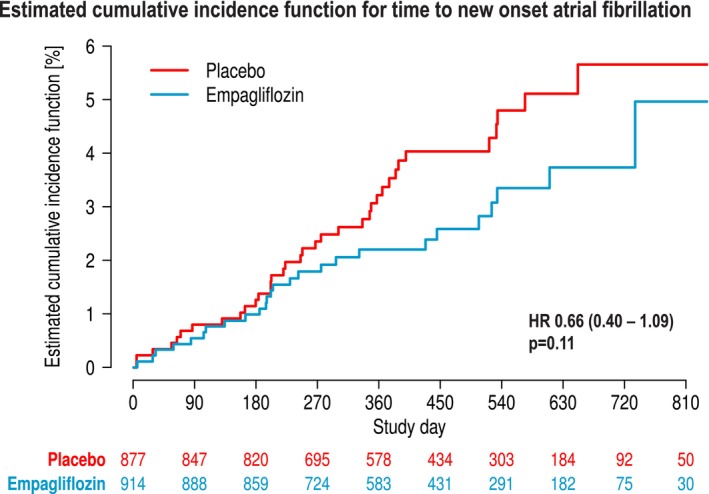
Cumulative incidence curve for the effect of empagliflozin and placebo on incident atrial fibrillation (adjusted for all‐cause mortality as competing risk). HR, hazard ratio.

Time to new‐onset AF was numerically reduced but formally not significantly different (HR 0.66, 95% CI 0.40–1.09, *p* = 0.11 for empagliflozin vs. placebo) (*Figure* *7*). Recurrent AF was not assessed.

### Safety assessments

The number of patients with any adverse event or any serious adverse event was in general higher in patients with AF than SR. Whereas the rate of any adverse event was lower in empagliflozin than placebo in those with SR and similar in both treatment groups in those with AF, the rates of any serious adverse event were lower in empagliflozin in both SR and AF patients. Adverse events leading to discontinuation were similarly distributed in empagliflozin and placebo. Symptomatic hypotension was more frequent in AF than in SR with no meaningful difference between empagliflozin and placebo. The same applies to acute renal failure with rather low event numbers (online supplementary *Table* *S2*).

## Discussion

This analysis of EMPEROR‐Reduced showed no significant differences in risk for HF hospitalization in patients with AF compared to SR. The eGFR decline was similar and baseline KCCQ‐CSS was lower in AF versus SR. There was no difference in the treatment efficacy of empagliflozin between AF and SR concerning the cardiovascular outcomes, speed of decline in eGFR and the improvement of KCCQ‐CSS. There was no significant but a numerical reduction of AF incidence (*Graphical Abstract*). The safety profile of empagliflozin was similar in AF and SR, while patients in AF revealed more adverse events.

Patients with baseline AF enrolled in EMPEROR‐Reduced had more severe HF outcomes, higher NT‐proBNP concentrations and worse renal function. The HF medication distribution was similar but there was fair treatment intensity with anticoagulants in patients with HF and AF of 85% compared to 11.7% in SR for most likely other indications. This is similar to previous reports (83.6% in AF vs. 13.1% in no AF).^11^ AF was not associated with higher event rates for the primary outcome neither when analysed with the prespecified model nor after adjusting for further risk factors. In the COMET trial, there was a positive association with clinical outcomes for patients with AF compared to SR, however, this effect disappeared after adjustment.^12^ Also in PARADIGM‐HF and ATMOSPHERE,^13^ while in the latter report new‐onset AF associated to higher event rates, which was not observed with permanent AF.^13^ The V‐HeFT study showed no association of AF with increased morbidity and mortality.^14^ Similar to our findings, the PRIME‐II study showed an association with overall mortality, which was not robust to multiple adjustments.^15^ The findings on clinical outcomes can be extended to the improvement of QoL as there was no interaction between the effects on KCCQ‐CSS and AF and SR. However, patients with AF had lower KCCQ‐CSS levels at baseline indicating that AF has an impact on QoL. Again, this has been shown in patients across age groups.^16^ These findings indicate that AF does not independently effect outcome rates in HFrEF but rather, since the associations are not robust after adjustments, may reflect a burden of confounders and factors leading to more complications in HFrEF.

The finding of the preserved treatment effect of empagliflozin in AF is reassuring as incidence and prevalence over time of AF in HF is high.^17^ In an analysis of 41 446 patients, the prevalence of AF increased from 53% to 60% in HFrEF and HF with mildly reduced ejection fraction and further to 65% in HFpEF.^18^ Consistently, the European Society of Cardiology Heart Failure Long‐Term Registry reports a slightly lower prevalence number in HFrEF with a trend to higher prevalence in HFpEF.^19^ In general, a higher age of AF patients compared to SR patients has been observed.^5^, ^10^, ^11^ However, also treatment effects according to age have been reported to be similar in HFrEF^20^ and HFpEF.^16^ Therefore, empagliflozin appears to work across rhythm groups and the age spectrum.

Impaired kidney function is one predictor of outcomes in HFrEF^21^ and treatment intensity and guideline‐directed medical therapy for HF is lower in patients with higher KDIGO classes.^22^ Empagliflozin reduces risk across all KDIGO risk categories.^23^ There was no interaction between the treatment effect of empagliflozin in each rhythm subgroup. This applies also to other risk indicators such as alcohol consumption, history of hypertension and diabetes, all associated with new‐onset AF and higher events rates.^2^, ^3^, ^5^, ^6^

In this study as in the previous analysis from DAPA‐HF (14% numerical reduction),^11^ there was no significant effect of empagliflozin to reduce incident AF (−34%, *p* = 0.11). In patients with diabetes, a cohort study provided evidence that sodium–glucose co‐transporter 2 (SGLT2) inhibitor treatment was associated with a lower risk of new‐onset AF compared to treatments with dipeptidyl peptidase‐4 inhibitors or glucagon‐like peptide‐1 receptor agonists amounting to 16–26%.^24^ This was recently confirmed in a HFrEF population.^25^ In diabetes patients at high cardiovascular risk undergoing AF catheter ablation, SGLT2 inhibitor treatment was associated with lower AF recurrences, less cardioversions and repeat ablations,^26^ which is similar to an analysis on HFrEF patients undergoing cryoballoon ablation with a higher freedom from AF recurrences.^27^ Incident AF is associated with increasing age^28^ and SGLT2 inhibitors exert cardioprotective effects involving cellular ageing pathways and autophagy.^29^ A reduction of filling pressure and subsequently less atrial wall stress could also be mechanistically important.^24^, ^25^, ^26^, ^27^ In a diabetes model, SGLT2 inhibition reduced AF duration and inducibility by involvement of sirtuin‐1 up‐regulation.^30^ The totality of data in diabetes and HF in this study as well as in mechanistic studies^11^ suggest an effect on incident AF. Low incidence of new‐onset AF, limited observation periods and the lack of continuous rhythm recordings in large outcome trials might have affected the robustness of this finding. Therefore, larger investigations over longer periods of time with rigorous continuous rhythm monitoring are needed.

### Limitations

Treatment was not randomized to rhythm groups at baseline and may be subject to invisible confounding. Due to low numbers, different types of AF (permanent, persistent, or intermittent) were not explored. Furthermore, separating this population by SR and AF and further in groups with different risk constellations rendered numbers lower with the consequence of a limited statistical power. Groups are not randomized and might be subject to residual confounding. The data on AF were investigator‐reported as continuous rhythm monitoring was not performed.

## Conclusion

Atrial fibrillation was not associated with higher rates of cardiovascular outcomes compared to SR after adjustment. Empagliflozin reduces the risk of HF events, eGFR decline and improves QoL similarly in AF and SR. Although there was a numerical albeit not significant decline in incident AF, this finding needs further exploration as the overall incidence was low (*Graphical Abstract*).

## Supporting information


**Table S1.** Baseline characteristics.

**Table S2.** Adverse events by treatment allocation and heart rhythm (AF or SR).
