# Supplementary material for: Heart failure outcomes and empagliflozin effects in patients with heart failure and reduced ejection fraction in sinus rhythm or atrial fibrillation: Data from EMPEROR‐Reduced
Source: Eur J Heart Fail. 2025 Sep 16;27(11):2218–28. doi: 10.1002/ejhf.70021 (PMC12765366; doi:10.1002/ejhf.70021)
Supplement: Supplementary file 1 — Table S1. Baseline characteristics. [file EJHF-27-2218-s002.pdf]

Supplement Table 1 - Baseline characteristics

	Atrial fibrillation (AF)		Sinus rhythm (SR)		Total		p-value
Number of patients	928		1857		2785		
Age (yrs) SD	69.5	10	64.2	11.2	65.9	11.1	
Sex [N (%)]							0.0013
Male	733	79.0%	1363	73.4%	2096	75.3%	
Female	195	21.0%	494	26.6%	689	24.7%	
Race [N (%)]							<0.0001
White	707	76.2%	1215	65.4%	1922	69.0%	
Black/ African−American	43	4.6%	140	7.5%	183	6.6%	
Asian	142	15.3%	400	21.5%	542	19.5%	
Other including mixed race	16	1.7%	79	4.3%	95	3.4%	
Missing	20	2.2%	23	1.2%	43	1.5%	
Region [N (%)]							<0.0001
North America	94	10.1%	144	7.8%	238	8.5%	
Latin America	278	30.0%	806	43.4%	1084	38.9%	
Europe	420	45.3%	509	27.4%	929	33.4%	
Asia	130	14.0%	253	13.6%	383	13.8%	
Other	6	0.6%	145	7.8%	151	5.4%	
LVEF							
<20	69	7.4%	163	8.8%	232	8.3%	0.2025
≥20 to ≤25	248	26.7%	520	28.0%	768	27.6%	
>25 to ≤30	331	35.7%	686	36.9%	1017	36.5%	
>30 to ≤ 35	215	23.2%	363	19.5%	578	20.8%	
>35	65	7.0%	125	6.7%	190	6.8%	
Baseline NT−proBNP [median pg/mL] [(Q1;Q3)]	2327	1491, 4064	1597	918, 3057	1864	1078, 3415	<0.0001#
Baseline systolic BP [mmHg] [mean, SD)	122.9	15.8	122.6	15.7	122.7	15.8	0.6177
Baseline systolic BP [mmHg] [N (%)]							
<110	213	23.0%	432	23.3%	645	23.2%	0.9758
≥110 to ≤130	437	47.1%	867	46.7%	1304	46.8%	
>130	278	30.0%	558	30.0%	836	30.0%	
Baseline diastolic BP (mmHg) (mean, SD)	75.4	11.3	74.0	10.6	74.5	10.9	0.0021
Baseline heart rate [bpm] [SD]	74.1	13.2	70.8	11.1	71.9	12.0	<0.0001
Baseline weight [kg] [SD]	81.3	19.3	76.5	17.7	78.1	18.4	<0.0001
Baseline BMI [kg/m^2] [SD]	28.4	5.6	27.5	5.3	27.8	5.4	<0.0001
Baseline KCCQ-CCS mean (SD)	68.6	22.6	72.7	21.7	71.3	22.1	<0.0001
BMI							
<30	603	65.0%	1320	71.1%	1923	69.0%	0.0010
≥30	325	35.0%	537	28.9%	862	31.0%	
Baseline eGFR (CKD-EPI) [$mL/min/1.73m^2$] [SD]	57.9	19.7	67.2	22.2	64.1	21.8	<0.0001
Baseline eGFR (CKD-EPI) [$mL/min/1.73m^2$] [N (%)]							
≥60	411	44.3%	1155	62.2%	1566	56.2%	<0.0001
<60	517	55.7%	700	37.7%	1217	43.7%	
Baseline eGFR (CKD-EPI) [$mL/min/1.73m^2$] [N (%)]							<0.0001
≥90	62	6.7%	330	17.8%	392	14.1%	
60 to <90	349	37.6%	825	44.4%	1174	42.2%	
45 to <60	264	28.4%	366	19.7%	630	22.6%	
30 to <45	197	21.2%	253	13.6%	450	16.2%	
<30	56	6.0%	81	4.4%	137	4.9%	
Missing	0		2	0.1%	2	0.1%	
Baseline urine albumin−to−creatinine ratio [mg/g] [N (%)]							
Normal (<30)	450	48.5%	1076	57.9%	1526	54.8%	<0.0001
Microalbuminuria (30 to ≤300)	366	39.4%	562	30.3%	928	33.3%	
Macroalbuminuria (>300)	106	11.4%	209	11.3%	315	11.3%	
Missing	6	0.6%	10	0.5%	16	0.6%	
KDIGO risk category							<0.0001
Low risk	214	23.1%	723	38.9%	937	33.6%	
Moderately increased risk	278	30.0%	524	28.2%	802	28.8%	
High risk	230	24.8%	353	19.0%	583	20.9%	
Very high risk	200	21.6%	247	13.3%	447	16.1%	
Missing	6	0.6%	10	0.5%	16	0.6%	
Baseline haemoglobin [g/dL] [SD]	13.84	1.70	13.68	1.61	13.73	1.64	0.0153

Supplement Table 1 - Baseline characteristics

	Atrial fibrillation (AF)		Sinus rhythm (SR)		Total		p-value
Atrial fibrillation or flutter at BL ECG							
No	288	31.0%	1857	100%	2145	77.00%	<0.0001
Yes	640	69.0%	0		640	23.00%	
History of atrial fibrillation or atrial flutter[1] [N (%)]							
No	0		1790	96.4%	1790	64.3%	
Yes	928	100%	66	3.60%*	994	35.7%	
Missing	0		1	0.1%	1	<0.1%	
Baseline HS Troponin T median [IQR]	23.1	16.1 - 35.5	19.9	13.3 - 31.6	21.2	14.1 - 33.0	<0.0001#
History of HHF (in the last 12 months)[2] [N (%)]	339	36.5%	549	29.6%	888	31.9%	0.0002
Cause of HF [N (%)]							
Ischemic	420	45.3%	1002	54.0%	1422	51.1%	<0.0001
Non−ischemic	508	54.7%	855	46.0%	1363	48.9%	
Diabetes at baseline [N (%)]							
Diabetic	439	47.3%	962	51.8%	1401	50.3%	0.0252
Non−Diabetic	489	52.7%	895	48.2%	1384	49.7%	
NYHA class at baseline [N (%)]							<0.0001
I / II	662	71.3%	1456	78.4%	2118	76.1%	
III//V	266	28.7%	401	21.6%	667	23.9%	
Alcohol status							0.0006
Does not drink alcohol	573	61.7%	1273	68.6%	1846	66.3%	
Drinks alcohol (no interference with participation)	314	33.8%	517	27.8%	831	29.8%	
Missing	41	4.4%	67	3.6%	108	3.9%	
Anti-thrombotic drugs	869	93.6%	1398	75.3%	2267	81.4%	<0.0001
Anticoagulants	789	85.0%	217	11.7%	1006	36.1%	<0.0001
Direct factor XA inhibitors	302	32.5%	62	3.3%	364	13.1%	<0.0001
Direct thrombin inhibitors	86	9.3%	8	0.4%	94	3.4%	<0.0001
Heparins	9	1.0%	11	0.6%	20	0.7%	0.2661
Vitamin K antagonists	396	42.7%	141	7.6%	537	19.3%	<0.0001
Drugs used in heart failure	928	100.0%	1855	99.9%	2783	99.9%	0.3173
ACE inhibitors/ARBS/ARNi	828	89.2%	1653	89.0%	2481	89.1%	0.8672
ACE inhibitors/ARBs*	661	71.2%	1333	71.8%	1994	71.6%	0.7599
ACE inhibitors	449	48.4%	857	46.1%	1306	46.9%	0.2655
ARBs*	217	23.4%	482	26.0%	699	25.1%	0.1400
ARNi	172	18.5%	341	18.4%	513	18.4%	0.9124
Beta-blockers	874	94.2%	1765	95.0%	2639	94.8%	0.3345
Diuretics	889	95.8%	1753	94.4%	2642	94.9%	0.1151
Mineralocorticoid receptor antagonists	636	68.5%	1385	74.6%	2021	72.6%	0.0007
Diuretics other than MRAs	831	89.5%	1577	84.9%	2408	86.5%	0.0008
Loop or high ceiling diuretics	809	87.2%	1522	82.0%	2331	83.7%	0.0004
Thiazides or low ceiling diuretics	67	7.2%	145	7.8%	212	7.6%	0.5810
Other diuretics	45	4.8%	58	3.1%	103	3.7%	0.0229
Cardiac glycosides	234	25.2%	224	12.1%	458	16.4%	<0.0001
(ACEi/ARB/ARNi)	828	89.2%	1653	89.0%	2481	89.1%	0.8672
(ACEi/ARB/ARNi) + (Beta-blocker/Ivabradine)	783	84.4%	1604	86.4%	2387	85.7%	0.1550
(ACEi/ARB/ARNi) + (Beta-blocker/Ivabradine) + MRA	556	59.9%	1215	65.4%	1771	63.6%	0.0044
