# Supplementary material for: Heart failure outcomes and empagliflozin effects in patients with heart failure and reduced ejection fraction in sinus rhythm or atrial fibrillation: Data from EMPEROR‐Reduced
Source: Eur J Heart Fail. 2025 Sep 16;27(11):2218–28. doi: 10.1002/ejhf.70021 (PMC12765366; doi:10.1002/ejhf.70021)
Supplement: Supplementary file 2 — Table S2. Adverse events by treatment allocation and heart rhythm (AF or SR). [file EJHF-27-2218-s001.pdf]

Supplement Table 2: Adverse events by treatment allocation and heart rhythm (AF or SR).

	**Empagliflozin (n=2996)**		**Placebo (n=2989)**	
	n (%)	Rate/100 py	n/N (%)	Rate/100 py
**Patients with any SAE**				
Sinus rhythm	332 (35.4)	37.2	403 (43.9)	49.7
Atrial fibrillation	206 (44.2)	47.6	226 (49.0)	57.7
**Patients with AE leading to discontinuation**				
Sinus rhythm	141 (15.0)	13.1	136 (14.8)	12.5
Atrial fibrillation	86 (18.5)	15.4	82 (17.8)	15.1
**Patients with any AE**				
Sinus rhythm	653 (69.6)	121.8	682 (74.4)	143.6
Atrial fibrillation	378 (81.1)	177.4	367 (79.6)	175.7
**Symptomatic hypotension***				
Sinus rhythm	34 (3.6)	3.2	34 (3.7)	3.2
Atrial fibrillation	27 (5.8)	4.9	30 (6.5)	5.7
**Acute renal failure**				
Sinus rhythm	70 (7.5)	6.7	79 (8.6)	7.6
Atrial fibrillation	49 (10.5)	9.2	59 (12.8)	11.7

* investigator reported

AF; atrial fibrillation, SR; sinus rhythm, AE; adverse events, SAE; serious adverse events

Adverse events (AEs) are shown up to 7 days after discontinuation of study medication. Acute renal failure was defined by MedDRA narrow SMQ “Acute renal failure”
